# Supraspinatus muscle elasticity measured with real time shear wave ultrasound elastography correlates with MRI spectroscopic measured amount of fatty degeneration

**DOI:** 10.1186/s12891-017-1911-8

**Published:** 2017-12-28

**Authors:** Fabian Gilbert, Detlef Klein, Andreas Max Weng, Herbert Köstler, Benedikt Schmitz, Jonas Schmalzl, Dirk Böhm

**Affiliations:** 1 Department of Trauma, Hand, Plastic and Reconstructive Surgery, Julius-Maximilians-University of Wuerzburg, Oberduerrbacherstr. 6, D-97080 Wuerzburg, Germany; 2 Ortho Mainfranken Wuerzburg, Bismarckstraße 16, D-97080 Wuerzburg, Germany; 3 Department of Radiology, Julius-Maximilians-University of Wuerzburg, Oberduerrbacherstr. 6, D-97080 Wuerzburg, Germany

**Keywords:** Rotator cuff, MRI, Ultrasound, Fatty degeneration, Shoulder surgery

## Abstract

**Background:**

Fatty Degeneration (FD) of the rotator cuff muscles influences functional and anatomical outcome after rotator cuff repair. The MRI based estimation of fatty degeneration is the gold standard. There is some evidence that Ultrasound elastography (EUS) can detect local differences of tissue stiffness in muscles and tendons. Shear-wave elastography (SWE) was evaluated to determine the extent to which shear wave velocity was associated with measures of fatty degeneration. MRI-spectroscopic fat measurement was used as a reference to quantify the amount of fat in the muscle belly.

**Methods:**

Forty-two patients underwent SWE of the supraspinatus muscles at its thickest diameter. After ultrasound evaluation an MRI-spectroscopic fat measurement of the supraspinatus muscle was performed using the SPLASH-technique. A gel filled capsule was used to locate the measured area in the MRI. The values of shear wave velocity (SWV) measured with SWE and spectroscopic fat measurement were correlated statistically using Pearson’s correlation test.

**Results:**

Correlation of the fat amount measured with MRI-spectroscopy and the SWV measured with SWE was ρ =0.82. Spectroscopic measured fat ratio of the supraspinatus muscle ranged from 0% to 77.41% and SWV from 1.59 m/s to 5.32 m/s. In 4 patients no sufficient SWE could be performed, these individuals showed a larger diameter of the overlying soft tissue. SWV measured with SWE showed a good correlation with MRI spectroscopic fat amount of the supraspinatus muscle.

**Conclusion:**

These preliminary data suggest that SWE may be a sufficient tool in detecting and estimating the amount of fatty degeneration in the supraspinatus muscle in real time. Large overlying soft tissue may be a limitation in performing sufficient EUS.

Ethical Committee Approval: Nr: 156/14 Date 12th August 2014.

Level of Evidence: III.

## Background

Fatty degeneration (FD) of the rotator cuff occurs after tendon rupture or nerve damage and affects the functional and anatomical outcome after rotator cuff repair [10, 31]. Preoperative detection and classification of FD is an essential part of planning operative rotator cuff repair. In case of advanced FD a rotator cuff repair has high rates of failure and leads to inferior clinical results [20]. FD is a non-reversible process even after successful rotator cuff repair [2, 12, 25]. In the clinical setting the MRI based modified Goutallier classification for t1-weighted oblique sagittal images is comonly used [9]. Conventional ultrasound has lately become a standard diagnostic tool for detecting rotator cuff tears due to its high sensitivity [11, 14, 21]. Conventional ultrasound techniques only allow a semi-quantitative grading of FD and is still considered an experimental modality [30].

Ultrasound elastography (EUS) is most often clinically used to evaluate hepatic, thyroid and breast pathologies [5, 8, 27]. Several studies described the usability of EUS for detecting changes in the musculoskeletal system, but there remains a lack of standardization using this technique for detecting musculoskeletal pathologies in the clinical setting. The use of EUS for musculoskeletal application has been reviewed extensively elsewhere [1, 3, 18].

**Fig. 1:**
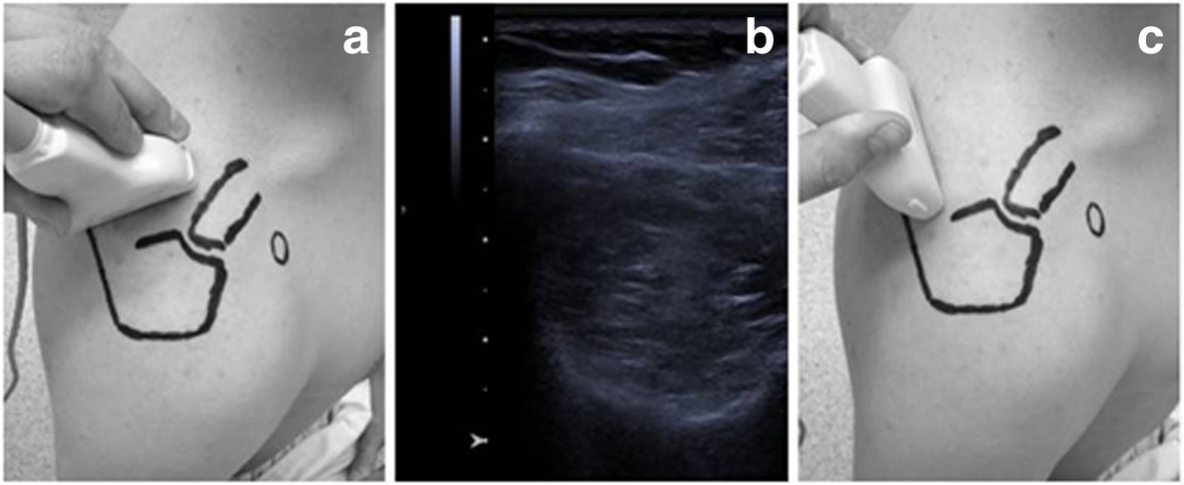
Experimental setting. The ultrasound probe was placed in an oblique sagittal position (**a**). The largest diameter of the supraspinatus muscles was detected using b-mode ultrasound (**b**). SWE measurement was performed at this location in a parallel orientation of the transducer to the muscle fibers (**c**). The velocity of shear wave propagation was measured at 10–15 points in the ROI. The measurement was repeated 5 times to cover almost the whole cross section area of the muscle. To ensure spectroscopic measurement at the same location the point of measurement was marked with a MRI visible gel capsule fixed to the patient’s skin

EUS includes different techniques (Fig. 1): Strain or compression elastography (SE) evaluates the elasticity of the underlying tissue by measuring the degree of deformation. Compression is applied manually or through physiological movements (breathing, vessel pulsation). This enables a semi-quantitative measurement of the tissue elasticity, as the elasticity is estimated by comparing the strain in the target tissue with strain of the surrounding tissue. Due to the manual applied pressure this technique has been shown to be operator depending [1, 3].

**Fig. 2:**
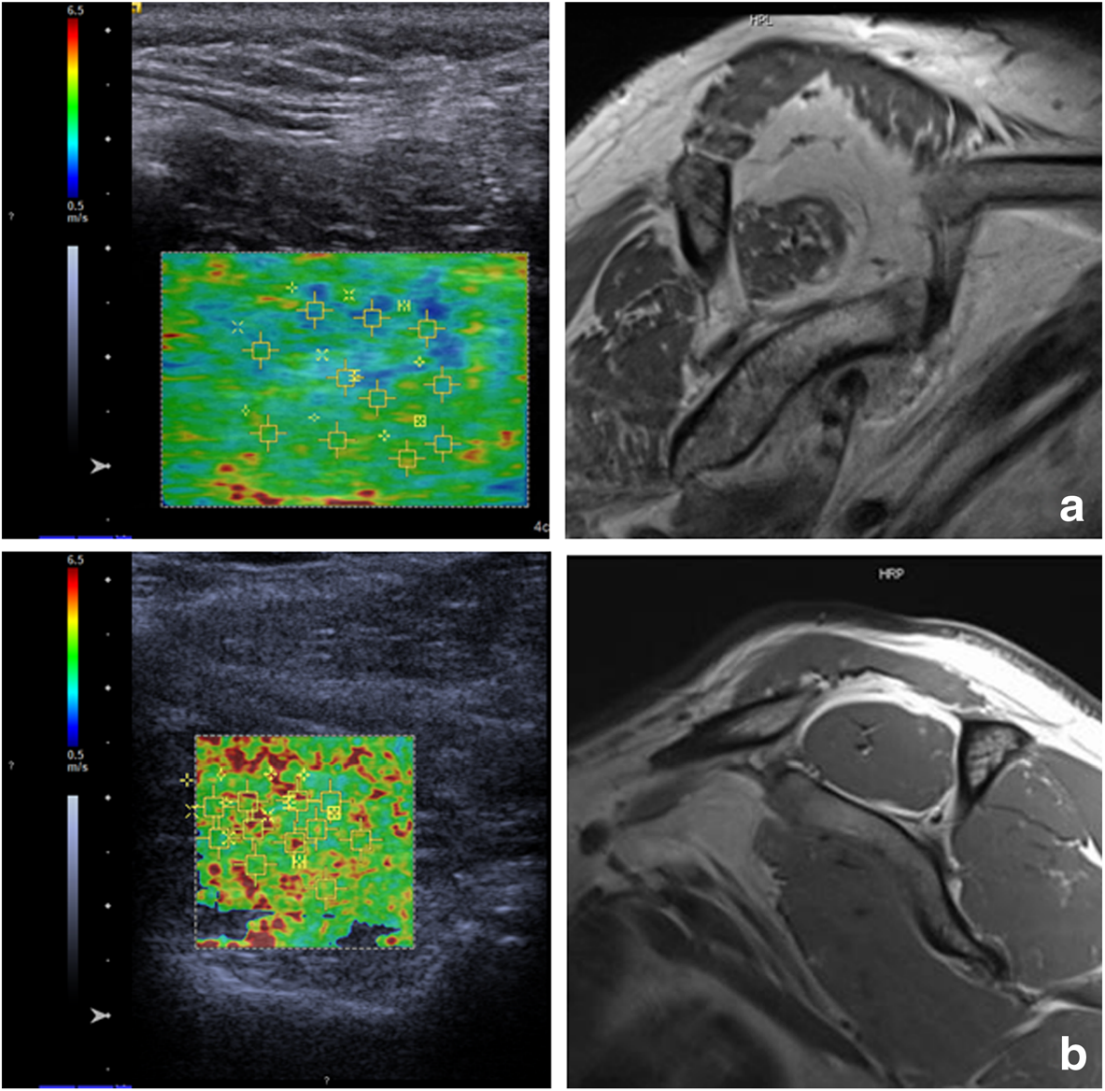
SWE for detecting FD in the supraspinatus muscle. The left column shows the color-coded shear wave velocity in the supraspinatus muscle. The right column shows the related T1 weighted oblique sagittal MRI images. Areas of higher fat amount appear blue to green in the shear wave EUS. Spectroscopic fat quantification with the SPLASH technique revealed a quantitative fat amount of (**a**) 77.41%, (**b**) 4.93%

Shear wave elastography (SWE) was introduced by Sarvazyan et al. in 1998. A radiation force of a focused ultrasonic push pulse (duration approximately 320 μs) is used to induce a tissue oscillation of up to 20 μm. These so-called shear-waves move transversally to the direction of the ultrasonic waves. The shear wave velocity (SWV) depends on the elasticity of the examined tissue [26]. Using tracking beams of the transducer (acoustic radiation force impulse, ARFI) the propagation velocity of the shear-waves can be measured. This method is propagated as an objective and reproducible quantification of the tissue elasticity which is in contrast to SE not depending from the manual compression by the observer (Fig. 2) [26]. However, as it is an ultrasound technique with manual application of the transducer it may be susceptible for observer dependent bias.

Experimental studies showed that SWE is capable of measuring muscle stiffness in the supraspinatus muscle and may detect regions of different elasticity in the muscle, without focusing on FD. These preliminary studies showed that the orientation of the ultrasound probe needs to be parallel to the muscle fibers to achieve reliable results for the tissue stiffness with SWE [4, 15].

The aim of this study was to evaluate how and if shear wave propagation trough the tissue of the supraspinatus muscle correlates with its amount of fatty degeneration. The semi-quantitative measurement using the Goutallier technique has been reported to have interobserver reliabilities between 0.43–0.86 [9, 24, 29]. In this study an experimental MRI-spectroscopic technique for fat measurement was used as reference. As this has been shown to allow an exact quantification of the FD in the supraspinatus muscle, by analyzing the ratio of the T1 related signal intensity of muscle and fat in a manually placed region of interest in a 2D MRI slice [16, 17]. In order to put these experimental methods in a clinical context, the results were correlated with the widely used MRI-based Goutallier scale. The hypothesis was that SWV correlates with the amount of fatty infiltration of the suprapinatus muscle tissue measured with a quantitative MRI spectroscopic method.

## Methods

### Level of evidence: III, analytic study

Institutional review board approval was acquired prior to the inception of this study, and informed consent was obtained from each patient.

**Fig. 3:**
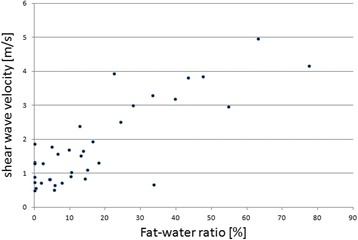
Scatter Plot of shear wave velocity [m/s] and amount of fat measured with MR-spectroscopy using the SPLASH technique [%]

Forty-two patients with history of rotator cuff tear were included in this study. Mean age of the patients was 58.8 ± 7,65 years (range from 40 to 76). Thirteen were female and 29 were male patients. Twenty-eight underwent rotator cuff repair, the time after surgery was 2.3 years (± 0.7). The other patients had a history of rotator cuff tear but were treated conservatively. Patients’ clinical features are shown in Table 1. The maximum diameter of the supraspinatus muscle was detected using conventional ultrasound (b-mode) and this area was marked using a gel capsule (Fig. 3). After detection of this area a shear wave ultra-sound measurement (SWE) of the supraspinatus muscle was performed with a Siemens Acuson S3000 ultrasound system (Siemens AG, Munich, Germany) using a linear transducer (10mHz) the SWV was assessed with the software of the ultrasound device (virtual touch tissue imaging quantification [VTIQ], Siemens Medical Solutions, Siemens AG, Munich, Germany). Tissue elasticity was measured by aligning the transducer parallel to the muscle fibers at the largest diameter of the supraspinatus muscle (Fig. 1). As the process of manually selecting data via the ultrasound system’s console has the potential to significantly introduce bias SWE was performed by one blinded radiologist (DK). The shear wave velocity (m/s) was measured at 10–15 points in the manually placed region of interest in 5 pictures trying to fully cover the muscles cross section. The forearm of the patients was placed on an armrest to avoid muscle contraction during the examination .

**Table 1:** Patients’ clinical features

Patients’ Features	
Total No. of patients	42
Sex, female:male	29:13
Age [years]	59.8 (±7.7)
Underwent rotator cuff repair	28
Time after surgery [years]	2.3 (±0.7)
Tendon retraction: Patte Classification I:II:III	18:17:7

**Fig. 4:**
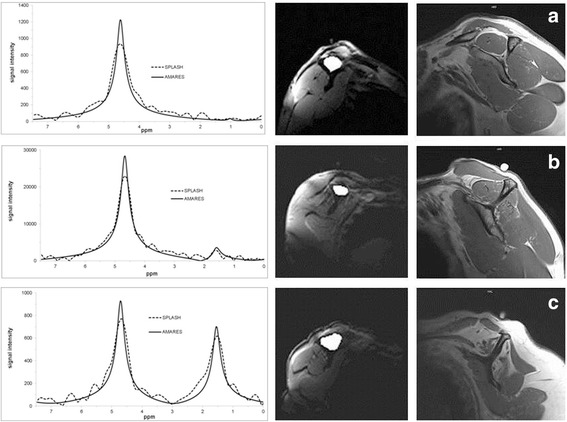
Spectroscopic analyses and quantification of the fat/water ration in the supraspinatus muscle (left column). The middle column shows the manual delineated borders of the M. supraspinatus in which the spectroscopic analyses were performed. The right collum shows the accordingly oblique-sagittal T1 weighted MRI images. Calculated values for these examples of the fat/water ratio were (**a**) 1.29%, (**b**) 12.67% and (**c**) 77.41%

On the same day a standard MRI scan of the shoulder was obtained. The MR-spectroscopy (SPLASH-technique (spectroscopic fast low angle shot)) and the postprocessing for quantification of the fat/water ratio was performed using a previously described technique by our workgroup [11, 17] (Fig. 4) Measuring the same area as in the ultrasound elastography was ensured with a gel capsule fixed to the patient’s skin.

All MRIs were rated according the MRI-based Goutallier Classification (oblique-sagittal t1-weighted images) by 5 raters. According to the scaling by Fuchs et al. Grade 0 was defined as no fatty infiltration, grade 1 as some fatty streaking of the supraspinatus, grade 2 as less fat than muscle, grade 3 as equal amounts of fat and muscle, and grade 4 as more fat than muscle [9]. All raters were orthopedic surgeons with clinical focus in shoulder surgery.

The thickness of the overlying tissue was directly measured in oblique-sagittal t1-weighted images using the clinical PACS System.

The correlation between the methods was calculated using Pearson’s rank correlation test (ρ). Degrees of reliability were set to the scale determined by Landis and Koch [19]. A statistical power analysis was performed using G*Power 3.1 [7]. Power analysis revealed a minimum number of Patients of *n* = 32 to obtain a power of 80% for finding a significant difference between a Pearson’s ρ of 0.65 an 0.85 (β > 0.8).

Median of the measured SWV of each muscle was statistically correlated with its spectroscopically measured fat amount using Pearson’s correlation test. The median of the Goutallier scale evaluated by independent 5 raters (with clinical focus on shoulder surgery) was correlated with the median of shear wave velocity and the spectroscopically measured fat amount using Spearman’s rank correlation test. Statistical analyses were performed using SPSS version 14 (IBM, Armonk NY, USA). Differences were considered as statistically significant, when *p* < 0.05.

## Results

Correlation of SWV measured with SWE in the patients’ supraspinatus muscles with the spectroscopic fat ratio showed a Pearson’s ρ of 0.82. This is a good correlation according to the criteria suggested by Landis and Koch [19]. Spectroscopic MRI analysis revealed fat ratios in 42 shoulders ranging from 0 to 77.41%, with a mean FD of 17.9% ± 18.9%. Medians of SWV ranged from 1.59 m/s to 5.32 m/s, with a mean of 1.81 m/s. SWV increased with higher fat water ratio. In four shoulders it was not possible to perform a sufficient SWE, consequently no SWE data were gained for these supraspinatus muscles. In these patients (*n* = 4) the mean diameter of overlying soft tissue was 20.04 mm (ranged from 11.47 mm to 31.44 mm). For the other patients (*n* = 38) the mean diameter of overlying soft tissue was 12.98 mm (ranged from 10.01 mm to 14.14 mm). The differences in the diameter did not reach the level of statistical significance.

Correlating the Goutallier scale with the spectroscopic fat measurement revealed a correlation of Pearson’s ρ =0.40 and with the SWV a correlation of ρ =0.48 (Table 2). Interpreted according to the criteria after Landis and Koch this represents a moderate agreement.

**Table 2:** Correlation and *p*-values of the different approaches to quantify FD

	R	*p*-value
Spectroscopy - Shear wave ultrasound	0.826	0.00008
Spectroscopy - Goutallier scale	0.404	0.008
Shear wave ultrasound - Goutallier scale	0.48	0.002

## Discussion

Fatty degeneration of a muscle cause changes of tissue elasticity [4, 22]. The aim of the current study was to test if shear wave velocity is affected by fatty infiltration of the supraspinatus muscle tissue. We observed an increased SWV when increased amounts of fat were found in the muscles tissue. Correlation of the spectroscopical measured fat amount with the clinical widely used Goutallier scale was only moderate.

Both shear wave elastography and MRI-spectroscopy represent experimental methods to quantify FD in the rotator cuff muscles. As the reliability of the MRI-based Goutallier scale is debatable and only represents a semi-quantitative classification system, objective methods which allow exact quantification are needed. Therefore, the present study compares two quantitative methods for measuring the FD even if spectroscopic fat measurement has no clinical relevance until yet, it is able to provide quantitative data of fat in a muscle. To allow clinical conclusions we compared the two experimental methods with the widely used semi-quantitative Goutallier scale. The relevance for using quantitative methods is highly indicated by the present data showing good correlation between the two quantitative approaches and only moderate correlation of these compared to the semi-quantitative Goutallier classification.

Regarding SWE there are no standard protocols for the application in the musculoskeletal system. Until now the most commonly applied technique in investigating the musculoskeletal system is strain elastography. As this is not a quantitative technique in regard to tissue elasticity, efforts towards establishing semi-quantitative scores have been made. Studies investigating the detection of FD with ultrasound or EUS are rare and the role for detection and the quantification of FD remains experimental. There are two studies comparing strain elastography (SE) to semi-quantitative MRI-analysis finding correlations between 0.744–0.81 [6, 28]. These studies are based on a different technique of elastography and are, due to the technique of SE, semi-quantitative analyses for the estimation of FD, therefore the comparability of these findings with the present study is limited, but the correlations reported seem to be comparable to the findings of this study.

Hatta et al. performed SE in 12 fresh porcine shoulders to investigate the influence of the overlying tissue to the strain ratio. They noticed that the strain ratio is influenced if the overlying tissue is larger than 22 mm [13]. In our study in 4 patients a sufficient SWE could not be achieved. If this was a technical problem or is in context with the large overlying soft tissue in these individuals remains unclear, as the difference of the diameter did not reach level of significance. But large overlying tissue might be a limitation for performing sufficient EUS.

SWE has been used experimentally for quantifying the mechanical properties of muscle tissue without focusing on the amount of FD. Itoigawa et al. measured the elastic module in different areas of human supraspinatus specimen. They found muscle elasticity was different with positioning of the transducer and proposed that SWE is able to quantify passive muscle stiffness [15]. Eby et al. confirmed these findings in a porcine brachialis muscle model and found that shear waves do not propagate well if the probe is not in an exact parallel position with the muscle fibers [4]. This resulted in parallel probe alignment to muscle fibers of the supraspinatus, but as ultrasound is a dynamic examination the correct alignment of the transducer may not be ensured for the whole procedure, which may represent a potential bias in the present investigation.

As only one blinded investigator (DK) performed SWE intra- or interobserver effects were not been evaluated in the present study and makes it susceptible to observer depended bias.

Muscle relaxation was not measured and was obtained by placing the arm on the chair’s armrest. The grade of muscle contraction highly affects the muscles stiffness [13]. This is supported by the findings of Muraki et al. who found significant changes in muscle stiffness with different levels of muscle contraction in a cohort of 23 healthy probands using SE [22].

Further limitations are that the ROI was selected individually in the supraspinatus muscle and there was no control if the whole muscle cross section area was covered, hence regional inhomogeneities may lead to incorrect values of overall shear wave propagation velocity [23]. Without having a biological reference (e.g. histological quantification) of the FD the exact quantification of the amount of FD remains unknown even with spectroscopic measuring techniques.

## Conclusion

The techniques used in this study are experimental and only few studies are comparable to the design of the present study. Further research must show the reproducibility of these results. We think estimation of FD in real time using SWE is feasible and may be a cost effective alternative to MRI quantification of FD. Especially when contraindications for the use of MRI are present or patients suffer from claustrophobia, this method may be an attractive alternative. According to our knowledge this is the first study trying to use quantitative methods estimating the amount of FD with ultrasound techniques. We think further basic science studies are necessary to create reference data to implement SWE into the clinical setting.

## Abbreviations

EUS: Ultrasound elastography
FD: Fatty degeneration
m/s: meters/s
mHz: mega Hertz
mm: millimeter
MRI: Magnet resonance imaging
PACS: Picture archiving and communication system
SE: Strain or compression elastography
SPLASH: Spectroscopic fast low angle shot
SWE: Shear-wave elastography
